# Virtually planned and CAD/CAM-guided secondary reconstruction of the mandibular condyle after malunion: from “unpredictable” to precise? —accuracy and outcomes

**DOI:** 10.1186/s40902-025-00497-2

**Published:** 2025-12-12

**Authors:** Paris Georgios Liokatis, Carl Peter Cornelius, Jens Tobias Hartung, Ina Dewenter, Wenko Smolka, Florian Andreas Probst, Philipp Poxleitner, Sven Otto, Katharina Theresa Obermeier, Yoana Malenova

**Affiliations:** Department of Oral and Maxillofacial Surgery and Facial Plastic Surgery, LMU Klinikum, Munich, Germany

**Keywords:** Condylar fracture, CAD/CAM, Accuracy, Virtual planning

## Abstract

**Background:**

In patients with malunited condylar fractures of the mandible surgical intervention is preferred when malocclusion and compromised masticatory function occur. This study evaluated the accuracy and clinical utility of virtual surgical planning (VSP) and CAD/CAM fabricated patient-specific implants (PSIs) for the secondary correction of post-traumatic mandibular condyle deformities.

**Methods:**

Accuracy of condylar segment repositioning was quantified by comparing the virtually planned joint surface with the actual postoperative same surface of the condylar head using Mimics and 3-Matic software (Materialise, Leuven, Belgium). Deviation was calculated by two methods: the Part Comparison Analysis (PCA) function of the analysis software and by measuring distances between four corresponding reference points on the planned and final segments.

**Results:**

The mean deviation between planned and final joint surfaces was 2.27 mm (range 4.69) with the PCA method and 2.56 mm (range: 8.99) with the reference-point method. No significant difference was observed between high and low condylar osteotomies. Accuracy was greatest along the lateromedial axis (1.83 mm; range: 6.98 mm) and lowest in the craniocaudal axis (3.30 mm; range: 6.75 mm). A good postoperative mandibular mobility was assessed in most cases with an average active mouth opening of 37 mm and no permanent sensory impairment was reported.

**Conclusion:**

Virtual surgical planning combined with CAD/CAM PSIs provides a reliable option for secondary reduction and fixation of condylar fragments, achieving high positional accuracy, good postoperative mandibular mobility, and a low rate of major complications.

## Introduction

Secondary reconstruction of the mandibular condyle following malunited fractures may be warranted over conservative management when patients present with facial asymmetry, malocclusion, restricted mandibular mobility, pain, or masticatory dysfunction [1, 2]. Clinical indicators favoring surgical intervention include a shortened mandibular ramus and, in particular, severe malocclusion [3]. Malocclusion is not only a prominent symptom but may also contribute to the development of other functional impairments, making it a key indication for surgical reconstruction of the temporomandibular joint (TMJ) [3].

Among the available surgical techniques, sagittal split osteotomy (SSO) of the ramus and subcondylar osteotomy (SCO) with reduction and osteosynthesis of the malunited fragment are most employed [2]. Many surgeons prefer SSO due to its predictability and familiarity [4, 5]. In contrast, SCO poses significant challenges, including accurately identifying the osteotomy site to mobilize the condyle-bearing fragment and repositioning it to restore ramus height—tasks that can be extremely difficult [3]. Achieving stable fixation adds to the complexity. These difficulties are further compounded by the small size of the condylar fragment, its intricate anatomical morphology, and potentially compromised bone quality. Moreover, if the proximal fragment remains dislocated outside the glenoid fossa for an extended period, intra- and periarticular soft tissue changes may make repositioning the condylar head extremely challenging and jeopardize its vascular supply [3].

There is growing interest in the potential of digital planning and patient-specific implants (PSIs) to improve the precision and predictability of secondary open reduction and internal fixation in the condylar region. These technologies have seen increasing adoption across various surgical disciplines, including reconstructive, orthognathic, traumatological, and craniofacial procedures [6, 7]. In maxillofacial trauma, virtual surgical planning and computer-aided design/computer-aided manufacturing (CAD/CAM) —based PSIs are well established, particularly in the treatment of complex midfacial and orbital fractures, where their accuracy is well documented [8, 9]. However, their application in mandibular fractures—especially those involving subregions of the condylar process (base, neck, head)—has remained relatively limited. Despite the higher associated costs, the clinical value, accuracy, and outcomes of these technologies in the management of condylar fractures have yet to be thoroughly validated.

The objective of this study is to evaluate the accuracy and clinical utility of virtual surgical planning and CAD/CAM PSIs in the secondary correction of post-traumatic deformities of the mandibular condyle. Furthermore, the study aims to assess the surgical workflow, associated complications, and overall clinical outcomes.

## Materials and methods

This retrospective study included patients who underwent secondary surgical reconstruction of malunited condylar fractures of the mandible. Secondary surgical treatment was defined as an intervention performed at least twelve weeks after the initial trauma. All patients were treated between 2014 and 2023 at the Department of Oral and Maxillofacial Surgery and Facial Plastic Surgery, University Hospital of LMU Munich.

Inclusion criteria required that patients had undergone secondary (at least 3 months after injury) reduction and osteosynthesis in the condylar region, guided by virtual surgical planning (VSP) and executed using CAD/CAM-manufactured tools.

The study was conducted in accordance with the STROBE guidelines for reporting observational studies [10]. Ethical approval was obtained from the institutional ethics committee (approval number 19-783), and informed consent was obtained from all participants.

### Digital planning, manufacturing of CAD/CAM implants

A high-resolution CT scan of the mandible and temporomandibular joint with a step of 0.625 mm and digital dental casts in occlusion were obtained to realize digital planning. The DICOM data from the CT scan were imported into the ProPlan CMF software (DePuy Synthes Maxillofacial, Paoli, CA / Materialise, Leuven, Belgium). Image processing involved converting the DICOM datasets into 3D surface models and removing soft tissue. Subsequently, an online planning session was conducted with the clinical engineer. If the initial fracture line could be identified, the osteotomy was planned along this line. A 3D-printed surgical guide was created to accurately define the osteotomy path and was also used for predrilling the screw holes required for implant fixation. The segment containing the condylar head was then repositioned to restore the vertical height of the mandibular ramus with reference to the opposite normal side. To determine the ideal final position of the proximal segment, the contralateral (intact) side was mirrored. If clinically relevant asymmetry between condyles or a fractured contralateral condyle rendered mirroring an inadequate criterion for defining ramus height, placing the affected condyle centrally within the glenoid fossa and achieving comparable joint space bilaterally was used as the main criteria. The mirrored model was then aligned within the glenoid fossa to ensure anatomical accuracy. Based on this reconstructed position, a patient-specific implant (PSI) was designed in various configurations to best support the restored anatomy.

### Surgical technique

A retromandibular or transoral approach was used for access to the condylar base, while a preauricular transparotid approach was preferred for osteotomies involving the condylar neck or head. In some cases, a combination of more than one approaches was necessary to allow adequate access for drilling and screw placement. Final fixation of the patient-specific implant was carried out under mandibulomaxillary fixation. Postoperatively, supportive therapy with intermaxillary elastics was applied as needed to assist in re-establishing proper occlusion.

All patients received physical therapy initiated in the first month after the operation with 2 sessions weekly for three to six weeks.

### Study variables, data acquisition, and analysis

Demographic information, medical history, clinical findings, and data related to digital planning and surgical treatment were retrospectively collected from patient records. The indication for surgery, as well as the time interval between the initial trauma and the surgical intervention, were also documented. The osteotomies were performed along the fracture line and classified as high osteotomies, corresponding to condylar head or neck fractures, and low osteotomies, corresponding to condylar base fractures, according to the AO-classification [11].

### Accuracy assessment

To quantitatively assess the accuracy of the bone segment reduction, the virtual surgical plan was compared with the postoperative CT data. Pre- and postoperative CT scans were acquired using the same device and imaging protocol. The postoperative CT DICOM files were segmented using a medical imaging software (Mimics, Materialise, Leuven, Belgium) to differentiate between soft tissue (HU < 300), bone tissue (HU 300–1500), and titanium (HU > 1500). Following segmentation, the data were converted into STL files. These STL models—representing the actual postoperative bone segments—were then compared with the STL files of the preoperative virtual plan using CAD analysis software (3-Matic, Materialise, Leuven, Belgium). For accurate comparison, the unchanged portions of the mandible—unaffected by surgery—were used for superimposition of the pre- and postoperative datasets. A fine-tuned superimposition was performed using the software’s semi-automatic global registration algorithm to optimize model matching and enabled the alignment of both datasets with a final precision of around 30 μm.

### Functional outcome

The functional outcome of the surgery was evaluated using the Helkimo mandibular mobility index, assessed both pre- and one year postoperatively [12]. Additional clinical parameters, including pain levels and occlusal status, were also recorded to support the functional assessment.

The accuracy was examined using two methods:

First, the function “part comparison analysis” (PCA) of the software (3-Matic, Materialise) was used to calculate the distance between the functional joint-bearing surface areas of the postoperative and planned mandibular condylar head. Only the upper surface of the condylar head was chosen due to its critical role in joint function and orientation of the repositioned segment within the glenoid fossa and to minimize a known disadvantage of the comparison algorithm, which calculates the distance between the closest and not the anatomically corresponding points of the two models leading to underestimation of the deviation [13]. This provided a surface deviation map of overall alignment accuracy.

Second, four standardized anatomical reference points were defined on the superior surface of the condylar head—specifically at the medial, lateral, anterior and posterior aspects. To ensure consistency, all reference points were selected by a single examiner [M.Y.]. The absolute linear distances between each pair of corresponding reference points on the planned and postoperative models were then measured according to the spatial axes (x-, y- and z-axis) using the same software, allowing for a precise evaluation of segmental repositioning accuracy. The x-axis represents transverse movement (lateral/medial), the y-axis represents sagittal movement (anterior/posterior), and the z-axis represents axial movement (cranial/caudal). The virtual planning model was selected as the static reference point.

### Statistical analysis

Statistical analysis was performed by Excel (Microsoft, Redmond, WA) and SPSS 26 (SPSS Inc., Chicago, IL). Statistical significance of the planning deviation between high and low subcondylar osteotomies was assessed using Friedman’s test. A p-value of < 0.05 was considered statistically significant.

## Results

Eight patients (3 males, 5 females) with a total of ten operated condyles were included in the study (Table 1). The median age was 49 years, and the median interval between trauma and surgery was 6 months. Four patients had previously undergone primary surgery that resulted in malunion or nonunion.

**Table 1:** Patient demographics, trauma characteristics, and treatment details

	Gender (M: male, F: Female)	Age	Leading Symptom(s)	Primary surgery performed	Active mouth opening (MO)	Delay until surgery (months)	Localization according to the AO-Classification (condylar head/neck/ base)	operated condylar fracture	Surgery duration (min)	Hospital stay (days)	Facial nerve damage (permanent or temporary)
1	M	34	Open bite	Yes	20	13	Neck/Head (bilateral)	Unilateral (with iliac crest bone)	330	7	None
2	F	40	Open bite	Yes	24	6	Base/Base (bilateral)	Bilateral	545	8	None
3	F	60	Occlusal interference joint pain	No	21	24	Head	Unilateral	195	6	None
4	M	14	Open bite, restricted mouth opening	Yes	26	3	Head	Unilateral	220	3	None
5	M	39	Open bite, restricted mouth opening	No	25	3	Head	Unilateral	190	9	None
6	F	59	Open bite, cross bite	Yes	27	5	Base/Base (bilateral)	Bilateral	320	8	None
7	F	62	Open bite, joint pain	No	19	10	Base	Unilateral	160	4	Temporary
8	F	64	Occlusal interference	No	22	6	Base	Unilateral	280	4	None

The median preoperative active mouth opening (MO) was 23 mm. The primary indications for referral and subsequent secondary reconstruction were malocclusion (most commonly anterior open bite and/or posterior premature contacts), functional impairment (limited mouth opening), and pain in the TMJ (Table 1).

The conservative measures attempted before referral to our department typically included analgesics/NSAIDs, soft diet, physiotherapy/jaw-motion exercises, occlusal adjustments and anti-inflammatory medication.

Six fractures were located in the condylar base according to the AO classification, and four in the condylar head or neck. Three patients presented with bilateral malunited condylar fractures; of these, two underwent surgery on both condyles, while one patient (Case 1) was operated on only one side, as satisfactory occlusion was achieved, allowing for conservative management of the contralateral malunited condyle. One patient (Case 1) required a bone graft from the iliac crest to bridge the defect after osteotomy and restoration of ramus height. The median operative time was 250 min, and the median hospital stay was 6 days.

At one-year follow-up, screw loosening with ectopic bone formation was observed in one case (Case 4). The median MO at one year postoperatively was 37 mm (Table 2). According to the Helkimo Index, dysfunction was classified as absent in four patients, mild in two, moderate in one, and severe in one (Case 4, with screw loosening and ectopic bone).

**Table 2:** Helkimo dysfunction index 1 year postoperatively

	Anamnestic Index	Clinical Index							Dysfunction score (0 = none, 1–4 = mild, 5–9 = moderate, 10–25 = severe)	Postoperative Complications observed
	Subjective Symptoms (none, mild, severe)	Pain during mandibular movement (none, mild, severe)	TMJ pain on palpation (none, mild, severe)	Muscle pain on palpation (none, mild, severe)	TMJ sounds (none, mild, severe)	TMJ Mobility				
						Impairement of active mouth opening (none : 35–45 mm, mild 25–35 mm, severe < 25 mm)	Laterotrusion impairment (none > 7 mm, mild 3–7 mm, Severe < 3 mm)	Protrusion (reduced < 3 mm, intermediate 3–7 mm, normal > 7 mm)		
1	None	None	None	None	None	None (42 mm)	None	None	None (0)	
2	None	None	None	None	None	None (41 mm)	None	None	None (0)	
3	None	None	None	None	None	None (37 mm)	Mild	Mild	Mild (2)	
4	Severe	Severe	Mild	Mild	None	Mild (30 mm)	Severe	Severe	Severe (14)	Ectopic bone, dislocation of osteosynthesis material
5	None	None	None	Mild	None	None (37 mm)	Mild	Mild	Moderate (6)	
6	Mild	None	None	None	Mild	None (33 mm)	Mild	Normal	Mild (3)	
7	None	None	None	None	None	None (38 mm)	None	None	None (0)	
8	None	None	None	none	None	None (39 mm)	None	None	None (0)	

Helkimo dysfunction index for each patient one year postoperatively. For the anamnestic index, patients receive 0 points for no symptoms, 1 point for mild symptoms, and 2 points for severe symptoms. For each parameter of the clinical index, patients receive 0 points for no dysfunction, 1 point for mild dysfunction, and 5 points for severe dysfunction. The final Helkimo score for each patient is shown in the shaded column

Seven of the eight patients were satisfied with the surgical outcome, reporting either no symptoms (*n* = 6) or only mild symptoms (*n* = 1). One patient developed postoperatively a temporary facial nerve weakness, which resolved spontaneously in three-months.

### 3D Mean surface distances

The mean deviation between planned and final position of the joint surface of all condylar segments was found to be 2.27 mm (range 4.69, IQR 1.87) with no statistically significant difference between high and low condylar fractures (Table 3).

**Table 3:** 3D median surface distances

	Osteotomy localization	Deviation		
		Median	Minimum	Maximum
1	High	1.15	0.0	3.08
2a	Low	2.26	0.0	4.87
2b	Low	2.44	0.0	4.72
3	High	2.29	0.0	5.04
4	High	0.85	0.0	2.39
5	High	5.54	0.0	9.56
6a	Low	0.87	0.0	3.73
6b	Low	3.68	0.0	8.62
7	Low	2.72	0.0	6.39
8	Low	1.37	0.0	2.65

3D median surface distances calculated with the Part Comparison Analysis (PCA) function of Materialise 3-matic for the upper portion of the condylar head. The PCA algorithm determines the median distance between the planned and the final model surfaces

### Absolute linear deviations

The overall median deviation in all unsigned axes for all points was 2.56 mm (range 8.99, IQR 2.82) and no statistically significant differences were observed between high and low osteotomies. For this reason, the findings are presented in Table 4 irrespective of the fracture type.

**Table 4:** Unsigned (absolute) linear deviations in the x-, y- and z-axis at four corresponding reference points

	x-axis (lateromedial)	y-axis (anteroposterior)	z-axis (craniocaudal)	All axes combined
Median	1.83	3.09	3.30	2.56
Interquartile range	2.17	4.63	2.01	2.82
Range	6.98	8.99	6.75	8.99

The median deviation in the x-axis was 1.78 (range 6.37, IQR 0.90) mm. In the y-axis 2.72 (range 5.70, IQR 4.64) mm and in the z-axis 3.11 (range 5.54, IQR 1.52) mm (Table 4). The Friedman’s test comparing the deviations between the three axes revealed a statistically significant lower deviation for the x axis compared to the other two (*p* = 0.002).

## Discussion

Delayed reconstruction of the condylar process following trauma presents a complex and challenging clinical scenario. Virtual planning and patient-specific implants (PSIs) have shown promising results in improving surgical outcomes in various regions of the facial skeleton [8]. This study aimed to evaluate the accuracy and clinical outcomes of these tools in subcondylar osteotomies.

### Accuracy of condylar reduction

Our findings indicate that the average deviation between the planned and actual postoperative position of the repositioned condyle was up to 2.56 mm with a maximum range of 8.99 mm. The greatest deviation occurred in the craniocaudal direction, with a median of 3.30 mm, followed by the anteroposterior axis at 3.09 mm. The best accuracy was achieved in the lateromedial direction, with a median deviation of 1.83 mm. No statistically significant differences were observed between high condylar neck osteotomies and low condylar base osteotomies.The wider per-axis ranges are explained by two outlier condyles (case 5 and the left condyle of case 6) with larger deviations. Regarding plausibility, the lower accuracy in these cases is consistent with intraoperative constraints: in case five, limited bone surface on the condylar head did not allow a precise positioning; in case six, compromised bone quality along the predrilled holes resulted in instable fixation.

When compared to digital planning and PSI-assisted procedures in other facial regions, the accuracy observed in condylar reconstructions was comparable to that reported for complex midface procedures [13]. However, a greater range of deviation was noted, particularly in the craniocaudal direction. Accuracy in the condylar region was found to be lower than in the orbital region [8]. Better accuracy outcomes, such as those reported by Schouman et al. [9] for the zygomatic bone, may be partly attributed to anatomical constraints in the condyle: the small size and complex curvature of the condylar segment reduce the contact area for surgical guides and implants. Additionally, the limited bone volume in the condylar head restricts the number of fixation screws that can be used for stable repositioning. Further complicating reconstruction are degenerative changes in the joint—such as alterations in the articular disc and surrounding soft tissues—which can hinder accurate repositioning of the condylar head within the glenoid fossa. Moreover, the condylar head is prone to arthritic degeneration, leading to compromised bone quality. These factors could also increase the risk of non-rigid fixation and subsequent displacement of the condylar head. Finally, some of the higher accuracy values reported for the zygoma [9]—or even for the condyle [14]—may reflect methodological differences, particularly the potential underestimation inherent in the Part Comparison Analysis algorithm used to align and compare models, as noted in the Methods section.

Nevertheless, given the TMJ’s capacity for functional adaptation [3], the principal benefit of virtual planning and PSIs is the reduction of variability, reliable restoration of ramus height and occlusion, and reproducible execution in defect cases, rather than pursuit of sub-millimetric accuracy per se.

### Implants design

**Fig. 1:**
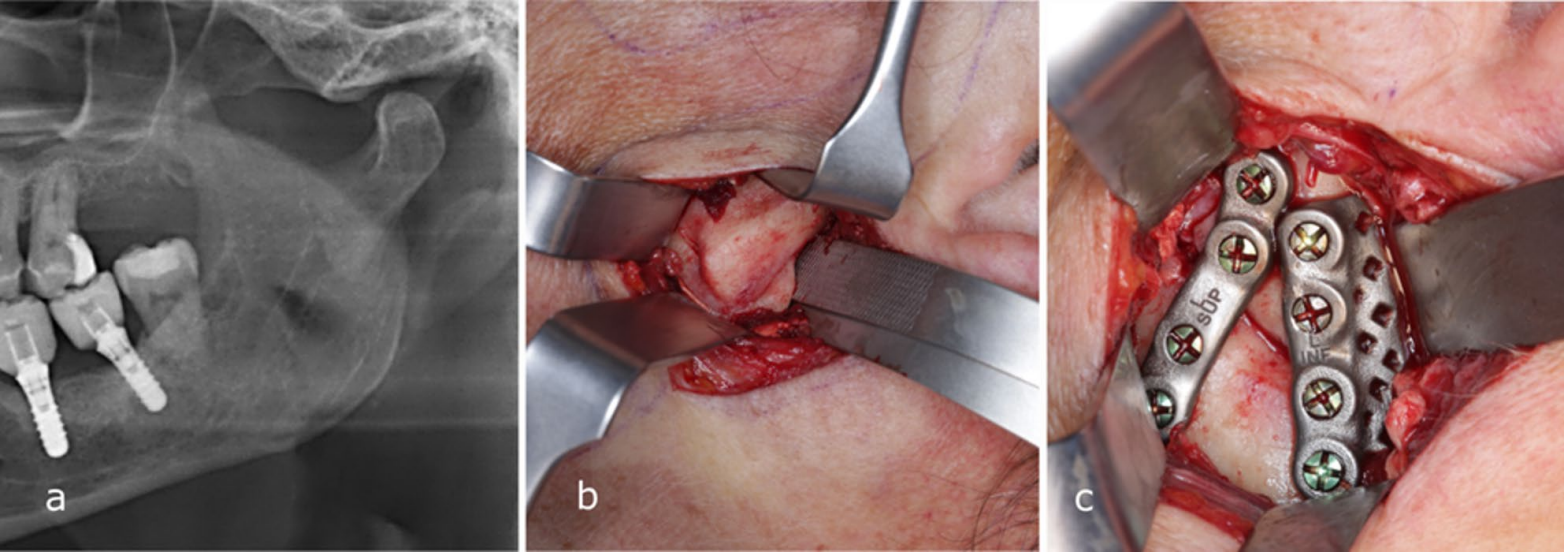
**a** Panoramic radiograph of patient 7 showing a laterally displaced, healed condyle following a condylar base fracture. **b** Intraoperative photograph of the laterally displaced condyle. **c** Intraoperative photograph after osteotomy, repositioning, and fixation with two patient-specific plates

**Fig. 2:**
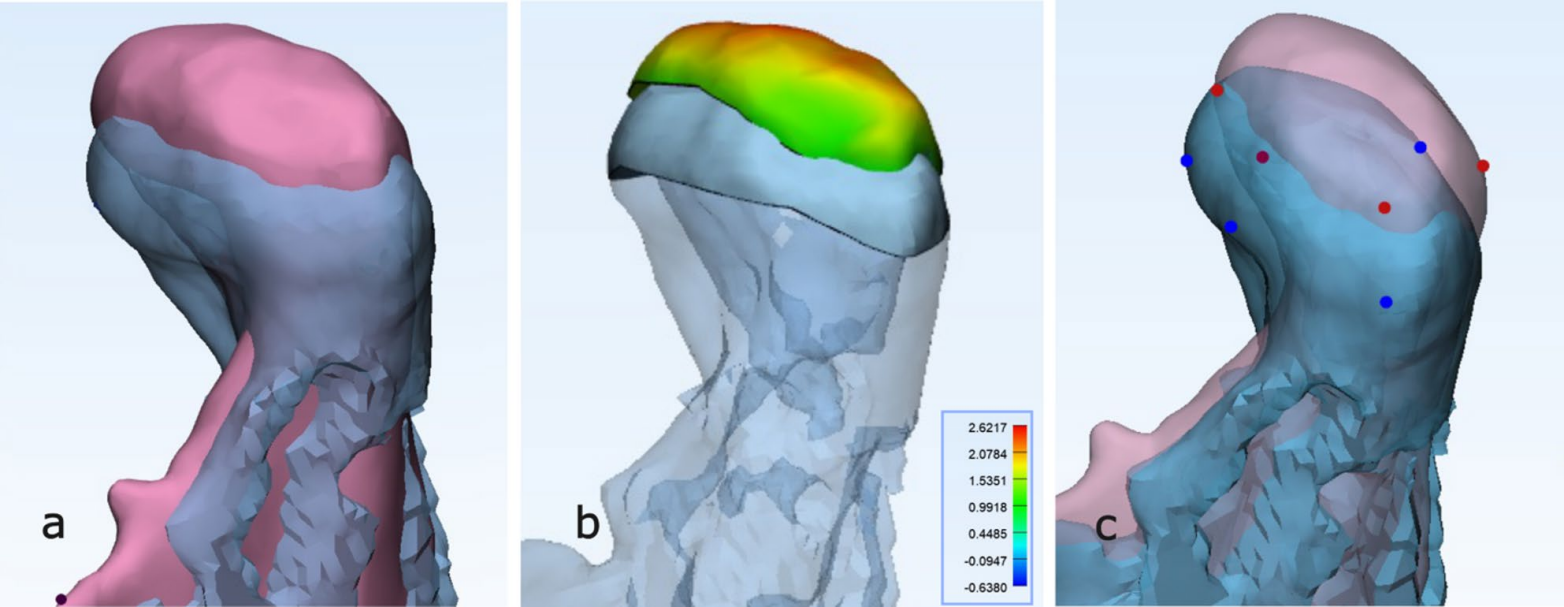
**a** Superimposed planned (pink) and postoperative (blue) condylar models of patient 7, showing the postoperative condyle positioned more caudally than planned. **b** Color-coded deviation map illustrating differences between planned and postoperative condyles, calculated with the Part Comparison Analysis (PCA) function of the analysis software. **c** Four corresponding reference points (red on the planned pink model and blue on the final blue model) marked on the upper condylar head region

An approach to improve accuracy in delayed reconstruction of the condyle after malunion may be the use of a single-plate, one-fit design, as proposed by other authors and supported by our own experience, provided that the specific characteristics of each case allow its application [14, 15]. Although secondary reconstruction performed after a long interval is inherently less accurate—due to bone remodelling and soft tissue changes as mentioned above—the use of a two-plate design (Figs. 1 and a-c and 2 and a-c) may introduce additional uncertainty and further compromise accuracy.

In our standard workflow, osteotomy guides define the target position of the condylar head via pre-drilled screw holes. In case 1 however, a segmental bone defect and a residual gap after repositioning left insufficient bony footprint on the condylar head for safe pre-drilling and guide fixation. Consequently, a single-plate strategy could not reliably control the three-dimensional position of the condylar segment.

We therefore employed a second plate to control and maintain the 3D position of the condylar head—height, anteroposterior and mediolateral translation, and rotation—in a centric position within the glenoid fossa until definitive fixation was achieved. The defect was reconstructed with an autologous iliac crest graft, which restored continuity and contributed to overall stability.

In summary, the “special” two-plate construct was a tailored solution to the biomechanical and reconstructive demands posed by the defect and limited bone stock, allowing stable, anatomically centered positioning of the condylar head.

### Clinical outcomes

While the Helkimo Index is useful for standardized reporting, its high sensitivity may overestimate the clinical relevance of minor or transient findings. In this study, 50% (4/8 patients) reported a CMD dysfunction at one year postoperatively. However, two of the four patients were classified as mild dysfunction, limited to slight restrictions in secondary movements (laterotrusion/protrusion) without reduced mouth opening or functional limitation. One patient with moderate dysfunction exhibited mild secondary-movement restriction with masticatory muscle pain, without major functional compromise and received a course of physical therapy leading to symptom remission. The remaining patient with severe dysfunction had displacement of osteosynthesis material and ectopic bone; symptoms resolved after hardware removal and debridement, indicating a treatable, device-related issue rather than a failure of the reconstructive concept. The remaining four patients were free of severe symptoms. Seven out of 8 patients expressed satisfaction with the operation outcome with satisfactory mandibular mobility and no dietary restrictions. The median active mouth opening was 37 mm (30–42 mm) for all 8 patients.

In their seminal 2013 publication [3], Ellis and Walker made recommendations for treating malunited condylar fractures, setting a relatively high threshold for surgical intervention and advocating intensive physical rehabilitation. While the benefits of physical therapy are well-established, such protocols can be demanding and may not be feasible for all patients. Success is highly dependent on patient compliance and lifestyle, which introduces variability and uncertainty in outcomes. Consequently, due to advances in digital planning and predictability of outcome there has been a growing trend in recent years toward surgical intervention [1, 16, 17].

### Subcondylar osteotomy vs. sagittal split osteotomy

Numerous surgical techniques have been proposed to address malocclusion resulting from malunited condylar fractures [16, 18]. Among these, the debate continues between two main approaches: open reduction and condylar reconstruction versus orthognathic surgery (including unilateral or bilateral sagittal split osteotomy [SSO], with or without Le Fort I osteotomy). Ellis and Walker favoured orthognathic surgery due to its higher predictability, particularly in cases presenting more than three months post-injury [3]. In such cases, anatomical and soft tissue changes around the joint complicate further osteotomy and reduction of the malunited condyle.

Conversely, Chen et al. compared these two approaches and found that SCO resulted in a greater increase in mouth opening— an average gain of 21.8 mm compared with 2.5 mm SSO [2]. This corresponded to a mean postoperative MO of 42.8 mm in the SCO group versus 35 mm in the SSO group. Although these differences did not reach statistical significance due to the small sample sizes (SCO: 8, SSO: 4), patients in the SCO group reported higher overall satisfaction. However, these favourable outcomes may have been influenced by factors other than the surgical technique itself, as SSO patients underwent surgery later (later than 18 months after fracture) compared with the SCO group (within 6 months), and the groups differed in their baseline MO (22 mm for SCO vs. 32 mm for SSO). Importantly, neither group experienced major complications such as facial nerve injury, infection, or the need for revision surgery. Based on these results, Chen et al. recommended subcondylar osteotomy over sagittal split osteotomy for post-traumatic correction.

**Fig. 3:**
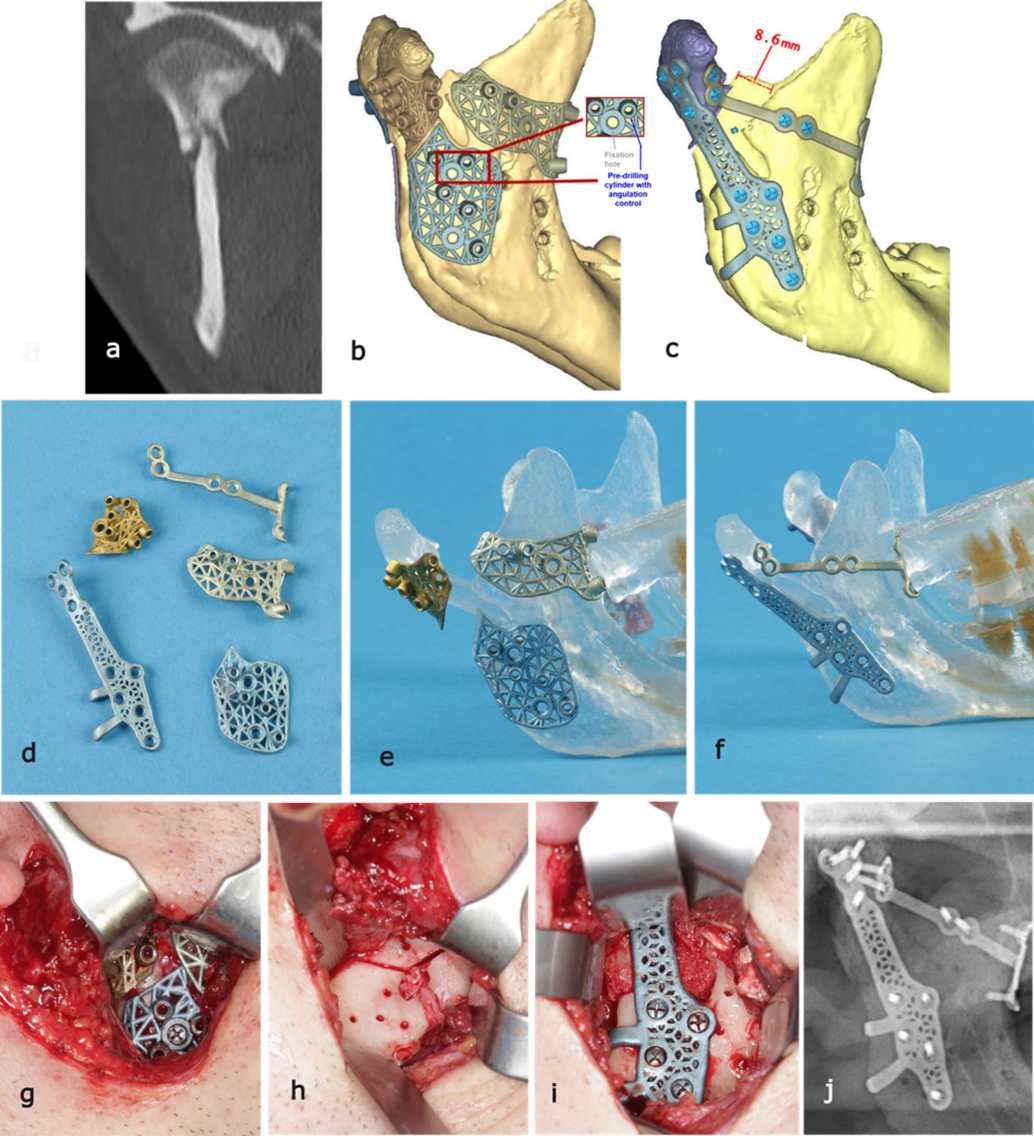
Demonstration of case 1, the most complex case in this series: **a** CT-scan showing a non-union with loss of ramus height in the right condyle after a base fracture. **b** Planned position of the patient-specific osteotomy and fixation guides. **c** Planned position of the patient-specific fixation implants. **d**-**f** Patient-specific armamentarium, including cutting/pre-drilling guides and fixation implants, displayed in their planned positions. **g**-**i** Intraoperative photographs showing, respectively, the cutting/pre-drilling guides in place, the completed osteotomy with pre-drilling, and the main PSI after fixation and transplantation of the iliac crest bone. **j** Postoperative panoramic radiograph showing the final position of the patient-specific implants.

**Fig. 4:**
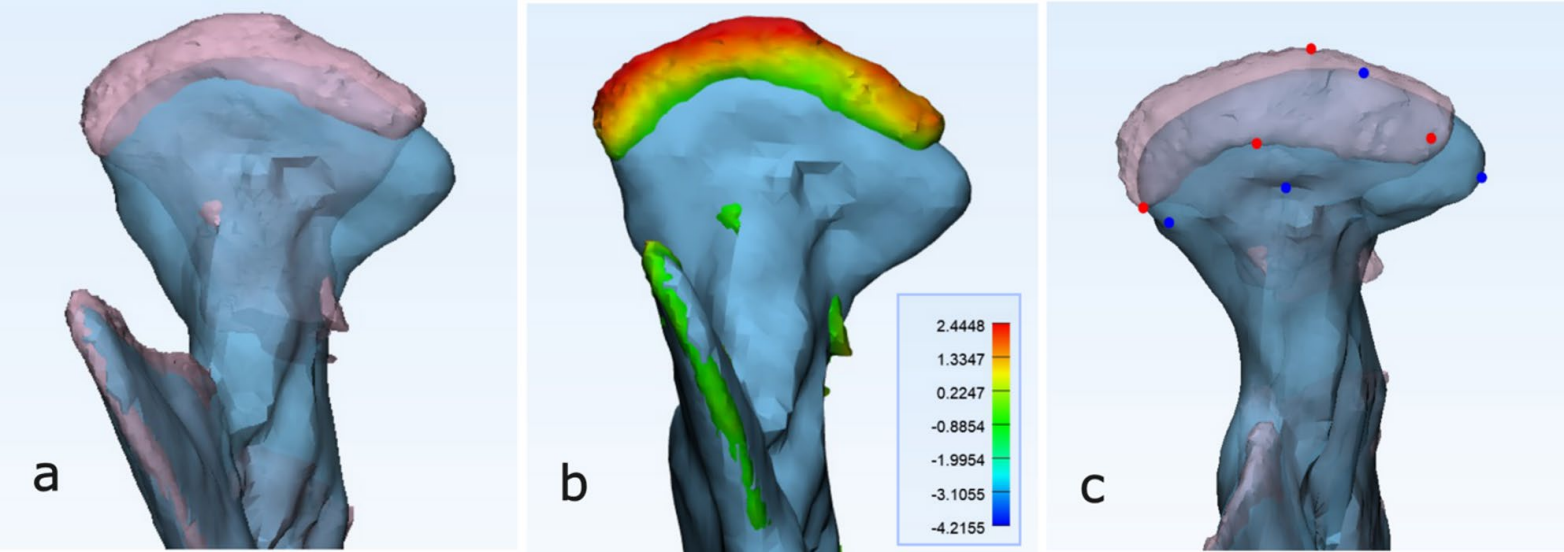
Analysis of case 1: **a** Superimposed planned (pink) and postoperative (blue) condylar models of patient 1, showing the postoperative condyle positioned more caudally than planned. **b** Color-coded deviation map illustrating differences between planned and postoperative condyles, calculated with the Part Comparison Analysis (PCA) function of the analysis software. **c** Four corresponding reference points (red on the planned pink model and blue on the final blue model) marked on the upper condylar head region

Our study supports the findings of Chen et al., particularly in terms of mandibular mobility, patient satisfaction, and a low incidence of severe complications. We demonstrate that digital planning and CAD/CAM implants enable accurate reduction and predictable outcomes, improving jaw function while potentially improving anatomical conditions for physiotherapy or even reducing the need for intensive and prolonged rehabilitation. Even in the most complex case in this study (Case 1; Figs. 3 and a-j and 4 and a-c), the patient-specific instrumentarium achieved an accuracy of better than 2 mm. However, a notable drawback of secondary open reduction and fixation in the condyle is the risk of screw loosening, which may necessitate re-operation. Our series confirms that even with PSIs, this risk cannot be eliminated.

Furthermore, it is important to note that orthognathic procedures are not necessarily less invasive or lower-risk than subcondylar osteotomies—particularly when performed bilaterally [19]. When combined with postoperative orthodontic treatment, they may significantly delay the patient’s return to normal function, increase costs and treatment duration.

Anatomical restoration of the condyle—as per the old surgical principle of “operate the defect”—may account for the relatively good postoperative mandibular mobility observed in our patients, even when surgery was performed long after the initial trauma (cases 1, 3, and 7). However, comparative data on mandibular mobility following SCO versus SSO remain limited. Moreover, in cases of a multi-fragmented condyle, performing an osteotomy with repositioning and fixation using a single patient-specific implant may be technically challenging or even impractical. Alternative PSI designs incorporating multiple components could offer a feasible option in such cases [20]. However, in such complex situations, placement of an alloplastic joint to replace the damaged condyle may represent the most viable solution [21].

Digital planning and CAD/CAM patient-specific implants entail additional up-front costs in the mid–four-figure range per case, depending on case complexity. Potential benefits include improved repositioning accuracy, a more efficient intraoperative workflow and shorter learning curve for the surgical team, and a reduced likelihood of reintervention for malposition or instability. A formal cost-effectiveness analysis was beyond the scope of this study and should be addressed in future work. In our cohort, postoperative dysfunction was limited to mild or moderate symptoms not requiring additional treatment in all but one patient; the single severe case was attributable to hardware displacement rather than failure of the reconstructive strategy. Overall, seven of eight patients reported satisfaction with the surgical outcome. These findings suggest that this technique has a justified role in the surgeon’s armamentarium when joint-preserving reconstruction is feasible.

### Limitations

The limitations of our study include the small sample size and absence of a control group using conventional osteosynthesis techniques. The follow-up period was limited to 1 year. While this interval is generally adequate to capture most postoperative complications, late events—such as progressive bone resorption or degenerative joint changes—may occur beyond 12 months. Longer-term follow-up is therefore warranted to fully assess durability and late complications. Future studies should consider including control cohorts treated with closed reduction or with bi- or unilateral SSO to provide robust comparative data.

## Conclusions

Virtual surgical planning and CAD/CAM implants offer a viable approach for secondary reduction and fixation of the proximal condylar fragment. They enable relatively high accuracy, favourable mandibular mobility, and a low risk of major complications, representing a promising alternative to conventional treatment methods for delayed condylar reconstruction.

## Data Availability

The data that support the findings of this study are not publicly available due to confidentiality reasons. The anonymized statistical data is available from the corresponding author (YM) upon reasonable request.
